# Supplementary figures and images for: Comparative transcriptome analysis of cold-tolerant and -sensitive asparagus bean under chilling stress and recovery
Source: PeerJ. 2022 Mar 22;10:e13167. doi: 10.7717/peerj.13167 (PMC8953502; doi:10.7717/peerj.13167)

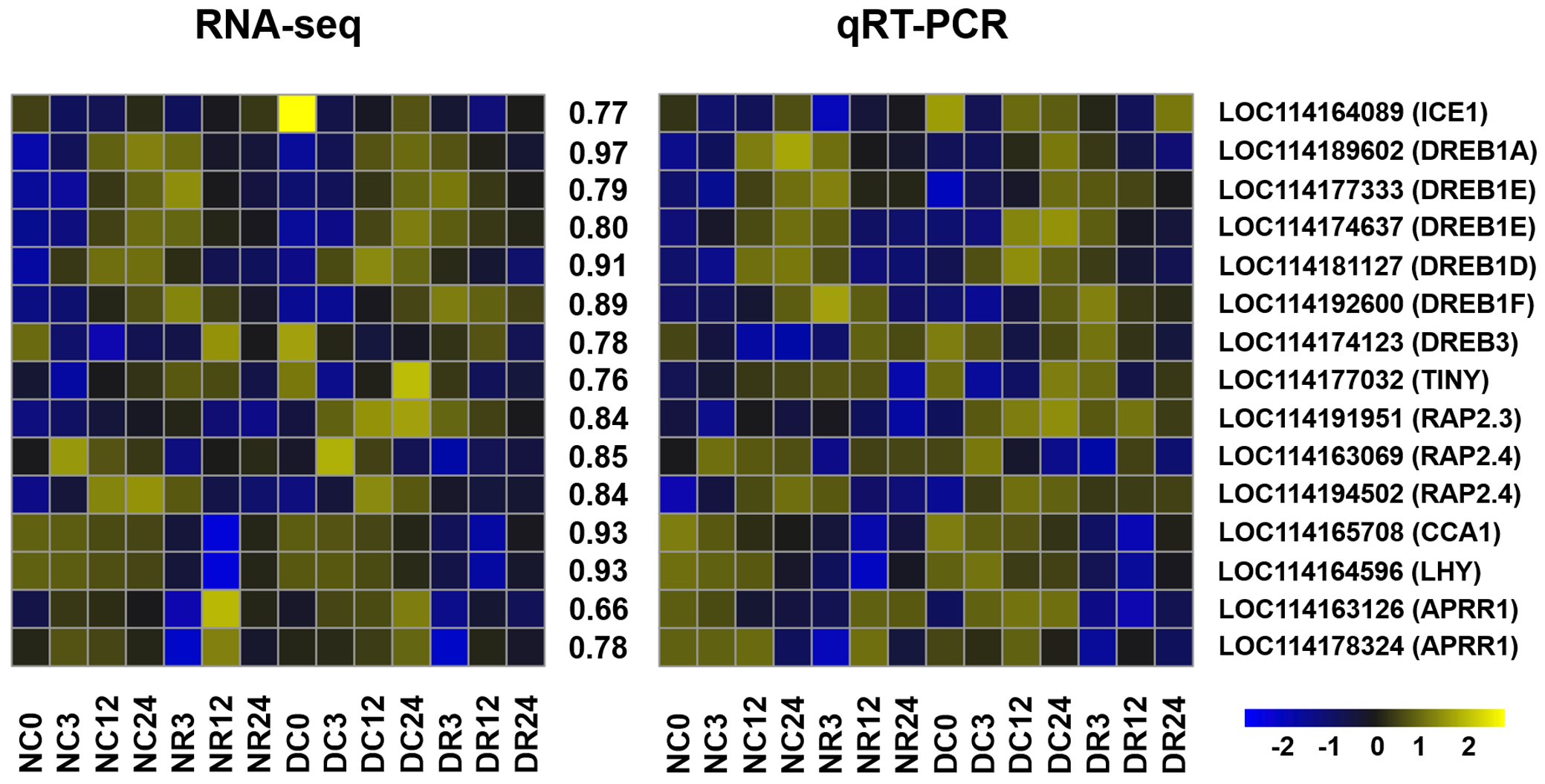

Supplement: Supplemental Information 1 — Heatmaps represent expression profiles of selected genes (labeled on the right side) obtained from RNA-seq (left) and qRT-PCR (right) analysis. The color scale at the bottom represents Z-score. The values between the two heatmaps represent the correlation between expression profiles of selected genes obtained from RNA-seq and qRT-PCR analysis. [file peerj-10-13167-s001.png]
