# Supplementary material for: Comparative transcriptome analysis of cold-tolerant and -sensitive asparagus bean under chilling stress and recovery
Source: PeerJ. 2022 Mar 22;10:e13167. doi: 10.7717/peerj.13167 (PMC8953502; doi:10.7717/peerj.13167)
Supplement: Supplemental Information 2 [file peerj-10-13167-s002.docx]

Table S1. Primers used for qRT-PCR validation of selected genes

| **Gene ID** | **Forward Primer** | **Reverse Primer** | **Size (bp)** | **Tm (^o^C)** |
| --- | --- | --- | --- | --- |
| LOC114164089 | GTAGCGTGACTGGGTTGGAT | GAAGAACCAGGCGGTGTAGA | 244 | 60 |
| LOC114189602 | CTGTCTCAATTTCGCCGACT | TCATCACTCTCGAAGCAACG | 243 | 60 |
| LOC114177333 | CAAATGCCAAGGAGATACGG | CCATGTTCTGAAGCCACTCA | 252 | 60 |
| LOC114174637 | GGGTTCCCAACAACAACAAC | GTTGGCTCACCTCTTCATCC | 231 | 60 |
| LOC114181127 | GCAGTAAGGCATCCCAAGAA | AAGTTGAGACAGGCGTACCG | 230 | 60 |
| LOC114192600 | TGAGACACGTCACCCTGTGT | CAGAGGAGGTAGCCGAGTTG | 236 | 60 |
| LOC114174123 | ATTTCCCCGAACTAGCCTCA | ATAACCAAAGCCACCTGCAC | 230 | 60 |
| LOC114177032 | CAACTCCGCCATTCTCAACT | TCGGCAGTTCCACTATCTCC | 239 | 60 |
| LOC114191951 | CGGTTACTCCCAAACCTCAA | TAGGCTCTCGCAGCTTCTTC | 231 | 60 |
| LOC114163069 | GGCTTAACTTCCCCAACCTC | ATGGTCTTGTCGTCCGAAAC | 238 | 60 |
| LOC114194502 | TCCCTCTCCCTCAACTTCCT | AAGTCGCCTCGGAGTCTGTA | 230 | 60 |
| LOC114165708 | CTGTCCTCCTGCATCTGTGA | ATGAACGGTAGCCTTTGGTG | 241 | 60 |
| LOC114164596 | TGCTACCTTTTGGCCCTATG | CCGTGTTGGATTTTGTGTTG | 240 | 60 |
| LOC114163126 | CCAGAGGTGGGATTGTCAGT | CAACCCCTTCAAAGTTGCTC | 241 | 60 |
| LOC114178324 | GTCTTCCCCAATGTCCTCCT | CTTTCTGCAAGCCGTTTTCT | 253 | 60 |
| LOC114185033 | AATGATCGGAATGGAAGCTG | TGGAATGTGCTGAGAGATGC | 264 | 60 |
