# Supplementary material for: Comparative transcriptome analysis of cold-tolerant and -sensitive asparagus bean under chilling stress and recovery
Source: PeerJ. 2022 Mar 22;10:e13167. doi: 10.7717/peerj.13167 (PMC8953502; doi:10.7717/peerj.13167)
Supplement: Supplemental Information 6 — N stands for Ningjiang 3 and D stands for Dubai bean. C0, C3, C12, C24 represent 5 ° C cold stress for 0, 3, 12, 24 h, respectively. R3, R12, R24 indicate 25 ° C recovery for 3, 12, 24 h, respectively. [file peerj-10-13167-s006.docx]

Table S5. Alignment result of illumina reads to reference genome. N stands for Ningjiang 3 and D stands for Dubai bean. C0, C3, C12, C24 represent 5℃ cold stress for 0, 3, 12, 24 hours, respectively. R3, R12, R24 indicate 25℃ recovery for 3, 12, 24 hours, respectively.

| **Sample** | **Total reads** | **Total mapped** | **Uniquely mapped** | **Reads map to '+'** | **Reads map to '-'** |
| --- | --- | --- | --- | --- | --- |
| DC0_1 | 47996844 | 45258437 (94.29%) | 42526461 (88.6%) | 21256625 (44.29%) | 21269836 (44.32%) |
| DC0_2 | 52365324 | 48956933 (93.49%) | 45933961 (87.72%) | 22964368 (43.85%) | 22969593 (43.86%) |
| DC0_3 | 48096036 | 44979144 (93.52%) | 42272770 (87.89%) | 21110375 (43.89%) | 21162395 (44%) |
| DC3_1 | 47652582 | 44616298 (93.63%) | 42091336 (88.33%) | 21015485 (44.1%) | 21075851 (44.23%) |
| DC3_2 | 55133016 | 51853559 (94.05%) | 49001178 (88.88%) | 24498183 (44.43%) | 24502995 (44.44%) |
| DC3_3 | 49559024 | 46492549 (93.81%) | 43958137 (88.7%) | 21976638 (44.34%) | 21981499 (44.35%) |
| DC12_1 | 49978882 | 46764336 (93.57%) | 44448504 (88.93%) | 22221933 (44.46%) | 22226571 (44.47%) |
| DC12_2 | 47942668 | 44650374 (93.13%) | 42420792 (88.48%) | 21206443 (44.23%) | 21214349 (44.25%) |
| DC12_3 | 47559354 | 44086119 (92.7%) | 41868079 (88.03%) | 20931505 (44.01%) | 20936574 (44.02%) |
| DC24_1 | 51815730 | 46493735 (89.73%) | 44455566 (85.8%) | 22212117 (42.87%) | 22243449 (42.93%) |
| DC24_2 | 46427652 | 42334450 (91.18%) | 40478777 (87.19%) | 20229861 (43.57%) | 20248916 (43.61%) |
| DC24_3 | 46618812 | 43380526 (93.05%) | 41558444 (89.15%) | 20777020 (44.57%) | 20781424 (44.58%) |
| DR3_1 | 51621116 | 48603738 (94.15%) | 47342416 (91.71%) | 23660226 (45.83%) | 23682190 (45.88%) |
| DR3_2 | 47647800 | 44602133 (93.61%) | 43420400 (91.13%) | 21701662 (45.55%) | 21718738 (45.58%) |
| DR3_3 | 50356508 | 47404382 (94.14%) | 46157842 (91.66%) | 23068998 (45.81%) | 23088844 (45.85%) |
| DR12_1 | 49281466 | 46332163 (94.02%) | 45095312 (91.51%) | 22533537 (45.72%) | 22561775 (45.78%) |
| DR12_2 | 51760632 | 48689474 (94.07%) | 47395647 (91.57%) | 23684458 (45.76%) | 23711189 (45.81%) |
| DR12_3 | 58841762 | 55336656 (94.04%) | 53838798 (91.5%) | 26901880 (45.72%) | 26936918 (45.78%) |
| DR24_1 | 44590028 | 42833680 (96.06%) | 41594527 (93.28%) | 20792251 (46.63%) | 20802276 (46.65%) |
| DR24_2 | 40545612 | 38914633 (95.98%) | 37789902 (93.2%) | 18892432 (46.6%) | 18897470 (46.61%) |
| DR24_3 | 49573732 | 47600464 (96.02%) | 46211577 (93.22%) | 23104736 (46.61%) | 23106841 (46.61%) |
| NC0_1 | 48949382 | 46378348 (94.75%) | 43974244 (89.84%) | 21982947 (44.91%) | 21991297 (44.93%) |
| NC0_2 | 55114076 | 52337203 (94.96%) | 49632551 (90.05%) | 24813138 (45.02%) | 24819413 (45.03%) |
| NC0_3 | 43759394 | 41489184 (94.81%) | 39314279 (89.84%) | 19655170 (44.92%) | 19659109 (44.93%) |
| NC3_1 | 43554522 | 40730469 (93.52%) | 38815553 (89.12%) | 19385825 (44.51%) | 19429728 (44.61%) |
| NC3_2 | 50472860 | 47869965 (94.84%) | 45612981 (90.37%) | 22803322 (45.18%) | 22809659 (45.19%) |
| NC3_3 | 58342724 | 54367494 (93.19%) | 51818914 (88.82%) | 25876096 (44.35%) | 25942818 (44.47%) |
| NC12_1 | 50719232 | 47548410 (93.75%) | 45136289 (88.99%) | 22569166 (44.5%) | 22567123 (44.49%) |
| NC12_2 | 42840560 | 40207445 (93.85%) | 38180808 (89.12%) | 19093607 (44.57%) | 19087201 (44.55%) |
| NC12_3 | 54410120 | 50861967 (93.48%) | 48360579 (88.88%) | 24184059 (44.45%) | 24176520 (44.43%) |
| NC24_1 | 45759822 | 41082778 (89.78%) | 38772152 (84.73%) | 19384915 (42.36%) | 19387237 (42.37%) |
| NC24_2 | 42532200 | 38029846 (89.41%) | 35851554 (84.29%) | 17925014 (42.14%) | 17926540 (42.15%) |
| NC24_3 | 42231026 | 39335438 (93.14%) | 37379471 (88.51%) | 18686590 (44.25%) | 18692881 (44.26%) |
| NR3_1 | 45428706 | 42880823 (94.39%) | 41778121 (91.96%) | 20882036 (45.97%) | 20896085 (46%) |
| NR3_2 | 50332616 | 47501471 (94.38%) | 46273210 (91.93%) | 23129523 (45.95%) | 23143687 (45.98%) |
| NR3_3 | 50014224 | 47241861 (94.46%) | 46021358 (92.02%) | 23001920 (45.99%) | 23019438 (46.03%) |
| NR12_1 | 51292914 | 48349203 (94.26%) | 47011113 (91.65%) | 23501667 (45.82%) | 23509446 (45.83%) |
| NR12_2 | 51098348 | 47995808 (93.93%) | 46681142 (91.36%) | 23335739 (45.67%) | 23345403 (45.69%) |
| NR12_3 | 46609350 | 43872029 (94.13%) | 42676870 (91.56%) | 21334013 (45.77%) | 21342857 (45.79%) |
| NR24_1 | 50639726 | 47982482 (94.75%) | 46502464 (91.83%) | 23243710 (45.9%) | 23258754 (45.93%) |
| NR24_2 | 52279112 | 49289933 (94.28%) | 47715693 (91.27%) | 23858809 (45.64%) | 23856884 (45.63%) |
| NR24_3 | 53236070 | 50060291 (94.03%) | 48431577 (90.98%) | 24216430 (45.49%) | 24215147 (45.49%) |

D stands for Duban bean, while N is short for Ningjiang 3. C0, C3, C12, C24 represent 5℃ cold stress for 0, 3, 12, 24 hours, respectively. R3, R12, R24 indicate 25℃ recovery for 3, 12, 24 hours, respectively.
