# Supplementary material for: Comparative transcriptome analysis of cold-tolerant and -sensitive asparagus bean under chilling stress and recovery
Source: PeerJ. 2022 Mar 22;10:e13167. doi: 10.7717/peerj.13167 (PMC8953502; doi:10.7717/peerj.13167)
Supplement: Supplemental Information 7 — N stands for Ningjiang 3 and D stands for Dubai bean. C0, C3, C12, C24 represent 5 ° C cold stress for 0, 3, 12, 24 h, respectively. R3, R12, R24 indicate 25 ° C recovery for 3, 12, 24 h, respectively. [file peerj-10-13167-s007.docx]

Table S6. Statistics of transcription factors upregulated in Ningjiang 3 and Dubai bean at all stages. N stands for Ningjiang 3 and D stands for Dubai bean.

C0, C3, C12, C24 represent 5℃ cold stress for 0, 3, 12, 24 hours, respectively. R3, R12, R24 indicate 25℃ recovery for 3, 12, 24 hours, respectively.

|  | **Ningjiang 3 (Up-regulated)** | | | | | | | | **Dubai bean (Up-regulated)** | | | | | | | |
| --- | --- | --- | --- | --- | --- | --- | --- | --- | --- | --- | --- | --- | --- | --- | --- | --- |
| **TF Family** | **NC0** | **NC3** | **NC12** | **NC24** | **NR3** | **NR12** | **NR24** | **N-Total** | **DC0** | **DC3** | **DC12** | **DC24** | **DR3** | **DR12** | **DR24** | **D-Total** |
| bHLH | 3 | 1 | 1 | 3 | 5 | 17 | 13 | **43** | 10 | 2 | 8 | 12 | 3 | 8 | 13 | **56** |
| NAC | 5 | 2 | 3 | 4 | 4 | 8 | 6 | **32** | 5 | 2 | 2 | 2 | 2 | 12 | 13 | **38** |
| C2H2 | 1 | 0 | 2 | 6 | 0 | 10 | 12 | **31** | 5 | 1 | 1 | 6 | 0 | 5 | 8 | **26** |
| MYB | 2 | 2 | 2 | 1 | 5 | 12 | 7 | **31** | 5 | 1 | 3 | 7 | 0 | 8 | 7 | **31** |
| WRKY | 1 | 1 | 2 | 5 | 4 | 4 | 10 | **27** | 10 | 3 | 4 | 5 | 2 | 15 | 6 | **45** |
| AP2/ERF-ERF | 1 | 0 | 1 | 7 | 6 | 5 | 6 | **26** | 6 | 1 | 5 | 6 | 2 | 12 | 14 | **46** |
| MYB-related | 3 | 1 | 1 | 3 | 3 | 7 | 8 | **26** | 6 | 2 | 3 | 1 | 2 | 11 | 5 | **30** |
| Others | 1 | 0 | 0 | 2 | 1 | 11 | 7 | **22** | 1 | 1 | 1 | 3 | 2 | 9 | 6 | **23** |
| bZIP | 3 | 1 | 2 | 3 | 2 | 4 | 4 | **19** | 4 | 0 | 0 | 5 | 3 | 13 | 10 | **35** |
| HB-HD-ZIP | 1 | 0 | 0 | 1 | 3 | 12 | 2 | **19** | 8 | 1 | 2 | 10 | 0 | 3 | 11 | **35** |
| AUX/IAA | 5 | 1 | 0 | 0 | 2 | 7 | 2 | **17** | 1 | 0 | 0 | 6 | 1 | 2 | 3 | **13** |
| GARP-G2-like | 2 | 0 | 1 | 2 | 0 | 4 | 8 | **17** | 3 | 3 | 4 | 5 | 1 | 9 | 7 | **32** |
| TCP | 3 | 1 | 0 | 1 | 0 | 6 | 6 | **17** | 1 | 0 | 0 | 0 | 0 | 0 | 2 | **3** |
| mTERF | 1 | 1 | 0 | 3 | 1 | 5 | 4 | **15** | 5 | 3 | 1 | 1 | 0 | 1 | 0 | **11** |
| DDT | 2 | 2 | 2 | 2 | 2 | 2 | 2 | **14** | 1 | 1 | 1 | 1 | 0 | 1 | 0 | **5** |
| MADS-MIKC | 2 | 2 | 2 | 2 | 2 | 2 | 2 | **14** | 1 | 0 | 0 | 1 | 0 | 0 | 0 | **2** |
| C3H | 0 | 0 | 1 | 2 | 0 | 6 | 3 | **12** | 2 | 0 | 0 | 1 | 0 | 5 | 4 | **12** |
| GRAS | 1 | 1 | 1 | 0 | 1 | 4 | 3 | **11** | 2 | 0 | 1 | 6 | 1 | 11 | 2 | **23** |
| SET | 0 | 0 | 0 | 1 | 0 | 5 | 5 | **11** | 5 | 0 | 3 | 4 | 0 | 0 | 3 | **15** |
| C2C2-Dof | 0 | 0 | 1 | 0 | 4 | 2 | 3 | **10** | 4 | 0 | 3 | 6 | 0 | 7 | 4 | **24** |
| Trihelix | 3 | 1 | 1 | 1 | 0 | 2 | 2 | **10** | 3 | 1 | 2 | 2 | 0 | 3 | 3 | **14** |
| C2C2-GATA | 0 | 0 | 0 | 1 | 0 | 4 | 4 | **9** | 1 | 0 | 0 | 0 | 0 | 2 | 1 | **4** |
| HB-other | 2 | 0 | 0 | 1 | 1 | 3 | 2 | **9** | 1 | 0 | 1 | 2 | 0 | 4 | 1 | **9** |
| AP2/ERF-AP2 | 1 | 1 | 1 | 1 | 2 | 1 | 1 | **8** | 3 | 0 | 0 | 3 | 1 | 1 | 3 | **11** |
| MADS-M-type | 1 | 1 | 1 | 2 | 0 | 1 | 2 | **8** | 1 | 0 | 0 | 0 | 0 | 0 | 0 | **1** |
| SRS | 1 | 1 | 0 | 1 | 0 | 3 | 2 | **8** | 0 | 0 | 0 | 0 | 0 | 0 | 0 | **0** |
| FAR1 | 1 | 0 | 0 | 1 | 0 | 4 | 1 | **7** | 0 | 0 | 0 | 0 | 0 | 0 | 0 | **0** |
| GNAT | 0 | 0 | 0 | 2 | 0 | 2 | 3 | **7** | 3 | 0 | 1 | 3 | 1 | 2 | 0 | **10** |
| HB-BELL | 1 | 0 | 0 | 1 | 0 | 3 | 2 | **7** | 2 | 0 | 0 | 3 | 0 | 0 | 3 | **8** |
| LOB | 1 | 0 | 1 | 0 | 2 | 3 | 0 | **7** | 0 | 0 | 0 | 1 | 0 | 2 | 1 | **4** |
| HB-KNOX | 1 | 0 | 0 | 1 | 0 | 2 | 2 | **6** | 0 | 0 | 0 | 1 | 0 | 0 | 1 | **2** |
| C2C2-YABBY | 1 | 1 | 1 | 0 | 0 | 2 | 0 | **5** | 1 | 1 | 1 | 1 | 0 | 1 | 2 | **7** |
| SBP | 0 | 0 | 0 | 0 | 0 | 5 | 0 | **5** | 2 | 0 | 2 | 2 | 1 | 1 | 0 | **8** |
| B3 | 0 | 0 | 0 | 0 | 0 | 3 | 1 | **4** | 3 | 1 | 1 | 3 | 1 | 2 | 4 | **15** |
| CSD | 1 | 1 | 1 | 0 | 1 | 0 | 0 | **4** | 0 | 0 | 0 | 0 | 0 | 0 | 0 | **0** |
| NF-YA | 0 | 0 | 0 | 0 | 0 | 2 | 2 | **4** | 0 | 0 | 1 | 0 | 0 | 0 | 0 | **1** |
| SNF2 | 0 | 0 | 0 | 0 | 1 | 1 | 2 | **4** | 6 | 0 | 0 | 4 | 0 | 2 | 0 | **12** |
| Tify | 0 | 1 | 1 | 1 | 0 | 1 | 0 | **4** | 0 | 0 | 0 | 0 | 0 | 2 | 1 | **3** |
| Whirly | 0 | 0 | 0 | 0 | 0 | 3 | 1 | **4** | 1 | 0 | 0 | 1 | 0 | 0 | 0 | **2** |
| ARID | 0 | 0 | 0 | 1 | 0 | 1 | 1 | **3** | 0 | 0 | 0 | 0 | 0 | 0 | 0 | **0** |
| B3-ARF | 0 | 1 | 1 | 0 | 0 | 1 | 0 | **3** | 3 | 0 | 0 | 4 | 0 | 1 | 6 | **14** |
| HMG | 0 | 0 | 0 | 0 | 0 | 1 | 2 | **3** | 2 | 1 | 0 | 2 | 0 | 1 | 1 | **7** |
| SWI/SNF-BAF60b | 0 | 0 | 0 | 1 | 0 | 2 | 0 | **3** | 2 | 0 | 0 | 3 | 0 | 0 | 1 | **6** |
| E2F-DP | 0 | 0 | 0 | 0 | 0 | 1 | 1 | **2** | 1 | 0 | 0 | 1 | 0 | 2 | 0 | **4** |
| HSF | 0 | 0 | 0 | 1 | 0 | 0 | 1 | **2** | 3 | 2 | 3 | 2 | 1 | 6 | 2 | **19** |
| Jumonji | 0 | 0 | 0 | 0 | 1 | 1 | 0 | **2** | 2 | 0 | 0 | 1 | 0 | 2 | 3 | **8** |
| LIM | 0 | 0 | 0 | 0 | 0 | 1 | 1 | **2** | 0 | 0 | 0 | 0 | 0 | 0 | 0 | **0** |
| PLATZ | 1 | 0 | 0 | 0 | 0 | 0 | 1 | **2** | 0 | 0 | 0 | 0 | 0 | 0 | 0 | **0** |
| TRAF | 0 | 0 | 0 | 0 | 0 | 1 | 1 | **2** | 1 | 0 | 0 | 1 | 1 | 4 | 1 | **8** |
| TUB | 0 | 0 | 0 | 0 | 0 | 2 | 0 | **2** | 1 | 0 | 0 | 0 | 0 | 2 | 1 | **4** |
| Alfin-like | 0 | 0 | 0 | 0 | 0 | 1 | 0 | **1** | 0 | 0 | 0 | 0 | 0 | 0 | 0 | **0** |
| C2C2-LSD | 0 | 0 | 0 | 0 | 0 | 1 | 0 | **1** | 0 | 0 | 0 | 0 | 0 | 0 | 0 | **0** |
| CAMTA | 0 | 0 | 0 | 0 | 0 | 1 | 0 | **1** | 0 | 0 | 1 | 1 | 0 | 1 | 0 | **3** |
| CPP | 0 | 0 | 0 | 0 | 0 | 1 | 0 | **1** | 2 | 1 | 1 | 2 | 0 | 0 | 0 | **6** |
| GARP-ARR-B | 1 | 0 | 0 | 0 | 0 | 0 | 0 | **1** | 0 | 0 | 0 | 0 | 0 | 1 | 0 | **1** |
| GRF | 0 | 0 | 0 | 0 | 0 | 1 | 0 | **1** | 3 | 1 | 1 | 3 | 0 | 0 | 2 | **10** |
| HB-WOX | 0 | 0 | 0 | 0 | 0 | 1 | 0 | **1** | 0 | 0 | 0 | 0 | 0 | 0 | 0 | **0** |
| IWS1 | 0 | 0 | 0 | 1 | 0 | 0 | 0 | **1** | 1 | 0 | 0 | 1 | 0 | 2 | 1 | **5** |
| NF-YB | 0 | 0 | 0 | 0 | 0 | 0 | 1 | **1** | 0 | 0 | 0 | 0 | 0 | 1 | 0 | **1** |
| NF-YC | 1 | 0 | 0 | 0 | 0 | 0 | 0 | **1** | 0 | 0 | 0 | 0 | 0 | 1 | 0 | **1** |
| OFP | 0 | 0 | 0 | 0 | 0 | 1 | 0 | **1** | 0 | 1 | 1 | 0 | 0 | 1 | 2 | **5** |
| VOZ | 0 | 0 | 0 | 0 | 0 | 0 | 1 | **1** | 0 | 0 | 0 | 0 | 0 | 0 | 0 | **0** |
| BBR-BPC | 0 | 0 | 0 | 0 | 0 | 0 | 0 | **0** | 0 | 0 | 0 | 0 | 0 | 1 | 0 | **1** |
| BES1 | 0 | 0 | 0 | 0 | 0 | 0 | 0 | **0** | 0 | 0 | 0 | 1 | 0 | 0 | 0 | **1** |
| C2C2-CO-like | 0 | 0 | 0 | 0 | 0 | 0 | 0 | **0** | 1 | 0 | 0 | 0 | 0 | 0 | 0 | **1** |
| DBB | 0 | 0 | 0 | 0 | 0 | 0 | 0 | **0** | 0 | 0 | 0 | 0 | 0 | 0 | 1 | **1** |
| DBP | 0 | 0 | 0 | 0 | 0 | 0 | 0 | **0** | 0 | 0 | 0 | 0 | 0 | 1 | 0 | **1** |
| GeBP | 0 | 0 | 0 | 0 | 0 | 0 | 0 | **0** | 0 | 0 | 0 | 0 | 0 | 1 | 0 | **1** |
| LUG | 0 | 0 | 0 | 0 | 0 | 0 | 0 | **0** | 1 | 0 | 0 | 0 | 0 | 0 | 0 | **1** |
| NF-X1 | 0 | 0 | 0 | 0 | 0 | 0 | 0 | **0** | 1 | 1 | 1 | 0 | 1 | 1 | 1 | **6** |
| PHD | 0 | 0 | 0 | 0 | 0 | 0 | 0 | **0** | 1 | 0 | 0 | 1 | 0 | 3 | 0 | **5** |
| Pseudo ARR-B | 0 | 0 | 0 | 0 | 0 | 0 | 0 | **0** | 0 | 0 | 0 | 0 | 0 | 1 | 0 | **1** |
| Rcd1-like | 0 | 0 | 0 | 0 | 0 | 0 | 0 | **0** | 0 | 0 | 0 | 1 | 0 | 0 | 0 | **1** |
| RWP-RK | 0 | 0 | 0 | 0 | 0 | 0 | 0 | **0** | 0 | 0 | 0 | 0 | 1 | 1 | 0 | **2** |
| zf-HD | 0 | 0 | 0 | 0 | 0 | 0 | 0 | **0** | 1 | 0 | 0 | 0 | 0 | 2 | 2 | **5** |
| Total | 54 | 24 | 30 | 65 | 53 | 196 | 150 | **572** | 138 | 31 | 59 | 138 | 27 | 190 | 162 | **745** |

D stands for Duban bean, while N is short for Ningjiang 3. C0, C3, C12, C24 represent 5℃ cold stress for 0, 3, 12, 24 hours, respectively. R3, R12, R24 indicate 25℃ recovery for 3, 12, 24 hours, respectively.
